# Deceleration Assistance Mitigated the Trade-off Between Sense of Agency and Driving Performance

**DOI:** 10.3389/fpsyg.2021.643516

**Published:** 2021-06-02

**Authors:** Wen Wen, Sonmin Yun, Atsushi Yamashita, Brandon D. Northcutt, Hajime Asama

**Affiliations:** ^1^Department of Precision Engineering, The University of Tokyo, Tokyo, Japan; ^2^Toyota Research Institute, One Kendall Square, Cambridge, MA, United States

**Keywords:** driving assistance, sense of agency, performance, joint-control, intention

## Abstract

Driving assistance technology has gained traction in recent years and is becoming more widely used in vehicles. However, drivers usually experience a reduced sense of agency when driving assistance is active even though automated assistance improves driving performance by reducing human error and ensuring quick reactions. The present study examined whether driving assistance can maintain human sense of agency during early deceleration in the face of collision risk, compared with manual deceleration. In the experimental task, participants decelerate their vehicle in a driving simulator to avoid collision with a vehicle that suddenly cut in front of them and decelerated. In the assisted condition, the system performed deceleration 100 ms after the cut-in. Participants were instructed to decelerate their vehicle and follow the vehicle that cut-in. This design ensured that the deceleration assistance applied a similar control to the vehicle as the drivers intended to, only faster and smoother. Participants rated their sense of agency and their driving performance. The results showed that drivers maintained their sense of agency and improved driving performance under driving assistance. The findings provided insights into designing driving assistance that can maintain drivers’ sense of agency while improving future driving performance. It is important to establish a mode of joint-control in which the system shares the intention of human drivers and provides improved execution of control.

## Introduction

Driving automation technology has been developing rapidly in recent decades. Although full driving automation has not been applied to personal-use vehicles, most vehicles manufactured in recent years have implemented automation and assistance functions (SAE International, 2014, 2018), such as cruise control, which automatically controls the speed of the vehicle; lane keep assist, which keeps the vehicle driving within a lane; and automatic emergency braking, which automatically applies emergency brakes when a high risk of collision is detected. These technologies greatly improve safety and the overall driving experience (Teoh and Kidd, 2017; Muslim and Itoh, 2020). For example, a public-road experiment showed that foresighted deceleration control ensured safer driving near blind points (Saito et al., 2021). However, as driving assistance systems become more advanced, drivers face a trade-off between sense of control and driving performance/safety.

Many recent studies have raised the issue of trade-off between system performance and driver engagement. Usually, the more reliable an autonomous driving system is, the less the driver might be engaged (Wen et al., 2019). For example, a recent study examined the impact of system performance on driver performance in collision detection and avoidance (Fu et al., 2020). Fu et al. (2020) designed three levels of sensitivity in an automatic emergency braking system. The less-sensitive condition missed half of potential collisions. The perfect condition detected all potential collisions. The over-sensitive condition raised several false alarms but also correct potential collisions. As a result, participants were the most engaged in driving and succeeded in completing the driving course without any collisions in the less sensitive condition. In contrast, numerous participants failed to respond to undetected risks in time and crashed in the other two conditions. This raises the following question: Should a driving assistance system purposively relinquish some control to humans to ensure human driver engagement? This arguably is an ineffective solution because it may reduce driving safety and increase drivers’ workload.

In the present paper, we focused on a cognitive trait called “Sense of agency.” Sense of agency refers to the subjective feeling of controlling external events through one’s intentions and actions (Gallagher, 2000). It influences humans’ motivation to control things and their consequential actions and decisions (Karsh and Eitam, 2015; Wen and Haggard, 2018; Wen et al., 2020). In the case of driving, it refers to a person’s belief or feeling that they are controlling the vehicle through their own actions. Sense of agency is considered a key cognitive trait in maintaining driving engagement (Victor et al., 2018; Wen et al., 2019). Research on human-machine interfaces has shown that humans become progressively more disengaged as more automation is employed (Berberian et al., 2012; Navarro et al., 2016; Victor et al., 2018; Wang et al., 2018; Yun et al., 2018). While driving assistance technologies can greatly improve driving performance, sense of agency is reduced by the interventions of these systems (Yun et al., 2018).

A recent review paper suggested that sharing intention between a human driver and a driving assistance system may be critical to solving the trade-off between driving automation and retaining sense of agency (Wen et al., 2019). In our previous study, simply applying driving automation considerably reduced sense of agency (Yun et al., 2018). In the present study, we hypothesized that if the intention of the driving automation system is consistent with the human driver’s intention, it will not reduce sense of agency while improving driving performance. Previous empirical studies in cognitive psychology indicate that sense of agency can be improved by external assistance when people achieve better task performance even when their actual control is weakened (Wen et al., 2015b; Inoue et al., 2017). Yet, these studies involved very simple tasks with only one or two types of actions (e.g., keypresses). It remains unclear whether, in a real driving scenario, it is possible to improve driving performance while not disturbing sense of agency with sharing control. If this hypothesis is valid, sharing driving intentions or goals between human drivers and driving assistance systems may be a possible design solution that maintains sense of agency and driver engagement without reducing the input of the driving system.

In the present experimental driving task, drivers’ vehicles were cut off by another vehicle, which suddenly decelerated. Such circumstances are linked with a high risk of collision because it is difficult for drivers to maintain safe inter-vehicle distance and speed control. In the present study, participants either drove the vehicle by themselves or received driving assistance without being made aware of the assistance. They were told to quickly slow down to avoid any collision. The cut-in vehicle did not stop during the simulation and participants were told to keep driving but maintain a constant inter-vehicle distance. In the assisted condition, deceleration assistance was activated 100 ms after the cut-in vehicle deceleration resulting in a rapid deceleration of the ego-vehicle to a matching speed. As the deceleration assistance was designed to employ similar actions to the vehicle as the participants would intend, it was expected to improve driving performance while not reducing sense of agency. In addition, we did not induce a condition in which the driving assistance system provides a solution that conflicts with the driver’s intention because it is widely established that feedback against one’s intention considerably reduces sense of agency (Sato and Yasuda, 2005). Instead of comparing the sense of agency between conflicting and consistent driving assistance, we focused on the sense of agency under driving assistance and manual driving. At last, a previous study showed that task difficulty influences the effect of assistance on the sense of agency (Wen et al., 2015b). Therefore, to examine whether the driving assistance system had a different effect on sense of agency and driving performance when task difficulty (i.e., risk of collision) varied, we included two risk scenarios: high risk vs. low risk.

## Materials and Methods

### Participants

Nineteen healthy male adults (mean age = 22.4 years, *SD* = 1.4 years) who held valid Japanese driver’s licenses were recruited using a university-wide social media advertisement. A power calculation was performed using the effect size of the difference in sense of agency between assisted and manual driving conditions from our previous study (Yun et al., 2018; Cohen’s *d* = 0.60). This indicated that a sample size of 17 would be sufficient to provide a power of 0.8 (with *α* = 0.05, one-tail dependent *t*-test). A power of 0.8 is frequently used in the prior power calculations of psychological studies (Cohen, 1988, 1992) and studies on the sense of agency (e.g., Sidarus et al., 2017; Ohata et al., 2020). The sample size of the present study is also comparable with many psychological studies on the sense of agency (Wen et al., 2015a,b, 2017; Christensen et al., 2019; Ohata et al., 2020). All the participants had normal or corrected-to-normal visual acuity. The study was approved by the ethics committee of the School of Engineering at the University of Tokyo. Written informed consent was obtained from all participants before their participation. Additionally, we did not control or select for participants’ gender, but the volunteers that applied to participate in this study were all male.

### Task

**Figure 1:**
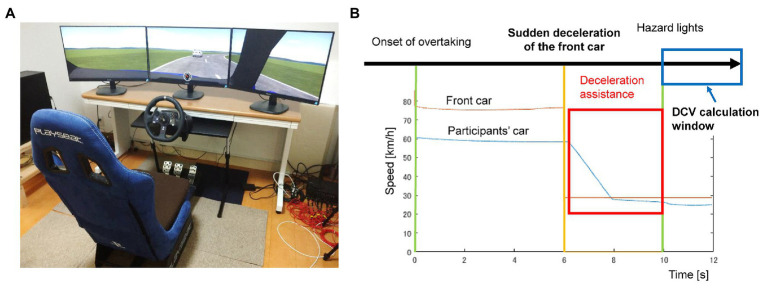
The experimental devices **(A)** and the timeline of the trial **(B)**.

The experiment was conducted using a driving simulator (CarSim, Mechanical Simulation Co.) in the laboratory (Figure 1). The experimental conditions were programmed in CarSim and MATLAB Simulink (2018b, MathWorks). A driving controller (Logitech G920) and three 27-inch LED monitors (273V7QDAB/11, Philips) were used to set up the driving environment (Figure 1A). The viewing distance to the central monitor was approximately 1 m, and the display had a horizontal angle of approximately 100° and a vertical angle of 19°.

Participants drove a simulated vehicle with an automatic transmission. In each trial, participants were instructed to drive their vehicle in the left lane (according to Japanese traffic laws) of a two-way road (one lane in each direction) at approximately 60 km/h. Other simulated vehicles were programmed to pass or overtake the ego-vehicle in the opposite lane. The width of each lane was 4 m. A few seconds after beginning each trial, a vehicle overtook the ego-vehicle and suddenly decelerated (without completely stopping) with blinking hazard lights (Figure 1B). The speed of the cut-in vehicle was 80 km/h before decelerating. The deceleration was instantaneous, although this is not possible in real life. However, this was merely designed to generate an emergency scenario, and no participant reported a sense of incongruity regarding the deceleration time of the cut-in vehicle. The distance between the ego-vehicle and cut-in vehicle was 30 m when the cut-in occurred. Participants were instructed to decelerate to avoid collision and to maintain safe inter-vehicular distance. They were instructed to maintain a constant speed with the ego-vehicle and a constant inter-vehicular distance. Another vehicle was programmed to drive in the opposite lane at the moment when the cut-in vehicle began decelerating. Therefore, the participants could not avoid the crash by steering to the opposite lane but had to decelerate. There were two deceleration conditions. In the high-risk condition, the speed of the cut-in vehicle dropped to 28.8 km/h instantaneously. This condition was linked to a high risk of collision if the participants’ car did not decelerate immediately. In the low-risk condition, the speed of the cut-in vehicle dropped to 46.8 km/h. In the low-risk condition, participants had more time to react to avoid the crash.

In the assisted condition, the system applied an automatic brake and decelerated the ego-vehicle to the speed of the cut-in vehicle (Figure 1B). The assistance started 100 ms after the sudden drop in the cut-in vehicle’s speed and ended 4 s after. During this assist, a simulated brake pressure of 4 MPa was continuously applied by the system when the speed of the ego-vehicle went higher than the cut-in vehicle, resulting in a deceleration of 4.98 m/s^2^. When the ego-vehicle’s speed was lower than the cut-in vehicle, no assistance was applied, and participants’ actual brake pressure was transmitted to the brake pedal. In addition, according to our pilot experiments, the reaction time of the human driver stepping on the brake pedal was approximately 700 ms. Therefore, the assisted condition ensured earlier deceleration and lowered the risk of collision. In other words, the deceleration assistance should have been activated in all the trials, and it only became inactive when the speed of the ego-vehicle decreased to a lower speed than that of the cut-in vehicle (even when still within the time-window of driving assistance).

Each trial lasted for 20–27 s, 10 s after the deceleration of the cut-in vehicle. Deceleration assistance was applied to all assisted conditions, even when the participants did not step on the brake pedal. The participants were not told about the type of trial (e.g., manual or assisted) they were assigned to in advance. They always began with manual driving, without knowing if the assistance system would be activated or not when encountering a cut-in vehicle.

In summary, there were two risk modes and two assistance conditions for each participant. The experiment was a full 2 × 2 (driving mode: manual vs. assisted × risk: high vs. low) design. Each condition was repeated 10 times, and the order of trials was randomized. In other words, the participants received deceleration assistance in 20 out of 40 trials, although they were not informed about the existence of the deceleration assistance system. After each trial, participants rated their sense of agency over their vehicle (“how much control did you feel you had over the speed of the vehicle?”) and the performance of their own driving (“how well you did drive the car and maintain inter-vehicle distance?”) with a 7-point scale (1 = not at all and 7 = a lot). Furthermore, participants’ actual driving performance was examined with two indices: the distance coefficient of variation (DCV) and speed coefficient of variation (SCV). DCV was the ratio of the standard variance of inter-vehicle distance to the mean inter-vehicle distance in the calculation window. DCV measured how well the inter-vehicle distance was kept constant (Uchiyama et al., 2003). SCV was the ratio of the standard variance of speed to the mean speed in the calculation window. SCV measured drivers’ ability to minimize their speed variance and maintain a constant speed. Smaller values of both indices indicate better driving performance. The calculation window was between 4 s after the onset of the cut-in vehicle’s deceleration and the end of the trial (Figure 1B). The time window of deceleration assistance did not overlap with the time window of performance (DCV and SCV) calculation. In other words, these two indices measured driving performance *after* the emergency deceleration rather than during the collision risk. These two indices were selected because participants were instructed to maintain both a constant speed with the ego-vehicle and a constant inter-vehicle distance. We did not analyze driving performance during the assistance because it ensured better performance (e.g., quicker and smoother deceleration) than the human drivers during the emergency deceleration. Instead, we predicted that the assistance would not only help prevent accidents but also improve driving performance after the deceleration was completed.

### Procedure

Experiments were conducted individually in a sound-attenuated chamber. Participants were first introduced to the driving simulator and were asked to drive in their usual manner. After receiving an explanation of the task, participants practiced for five or more trials without the deceleration assistance (i.e., under the manual condition) until they orally reported that they felt confident driving in the simulator. After practice, each participant conducted 40 actual trials with 5 min breaks after every 10 trials. After each trial, the driving view was turned off, and the two rating questions (agency rating and performance self-rating) were presented on the screen. The participants orally reported a number as their response to each item. The experiment took approximately 100 min per participant. No participant reported motion sickness during the experiment.

## Results

### Subjective Rating of Agency and Performance

**Figure 2:**
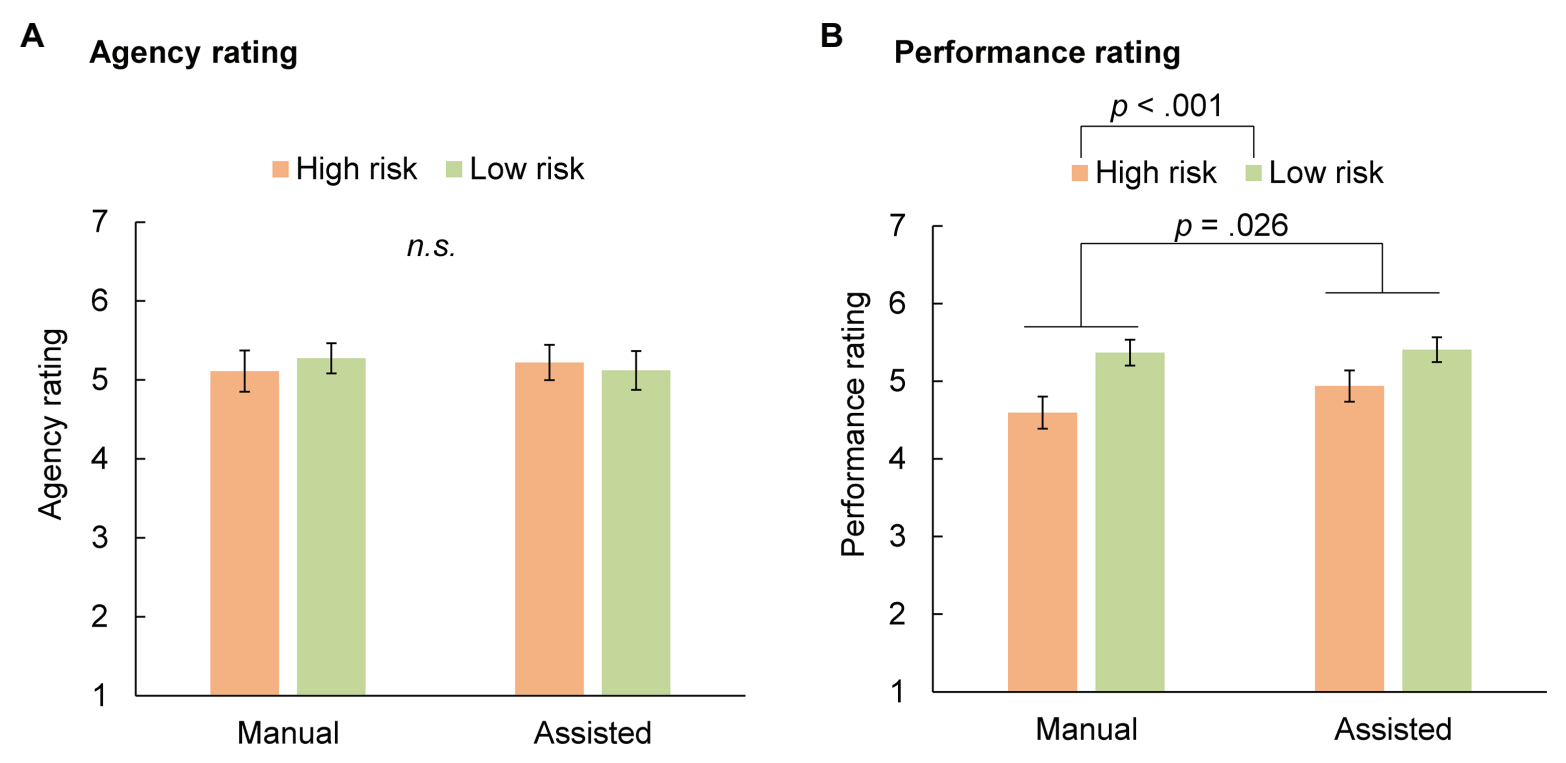
Average ratings of sense of agency **(A)** and driving performance **(B)**. The error bars represent the SEs.

Figures 2A,B show the drivers’ average agency and performance ratings in each condition, respectively. We used a 2 × 2 (driving mode: manual vs. assisted × risk: high vs. low) repeated measures ANOVA to examine the effect of the experimental conditions. Regarding the agency rating, the main effect of driving mode [*F*(1, 18) = 0.020, *p* = 0.888, *η*^2^_p_ = 0.001], risk [*F*(1, 18) = 0.017, *p* = 0.897, *η*^2^_p_ = 0.001], and the interaction between driving mode and risk [*F*(1, 18) = 1.280, *p* = 0.273, *η*^2^_p_ = 0.066] were all non-significant. The participants felt they had good control (rating > 5.1 on a 1–7 point scale) over the vehicle in all the conditions, regardless of whether they received assistance.

Regarding the subjective rating of driving performance, the main effect of the driving mode [*F*(1, 18) = 5.909, *p* = 0.026, *η*^2^_p_ = 0.247], and risk [*F*(1, 18) = 30.106, *p* < 0.001, *η*^2^_p_ = 0.626] were both significant. The interaction between driving mode and risk was non-significant [*F*(1, 18) = 4.091, *p* = 0.058, *η*^2^_p_ = 0.185]. The participants rated their driving performances higher when the collision risks were lower, and when they received driving assistance. The performance rating differed significantly between the manual and assisted conditions, indicating that the participants felt some difference between the two conditions. However, they attributed their driving performance to their own skills to the same extent across all the conditions.

### Actual Performance

**Figure 3:**
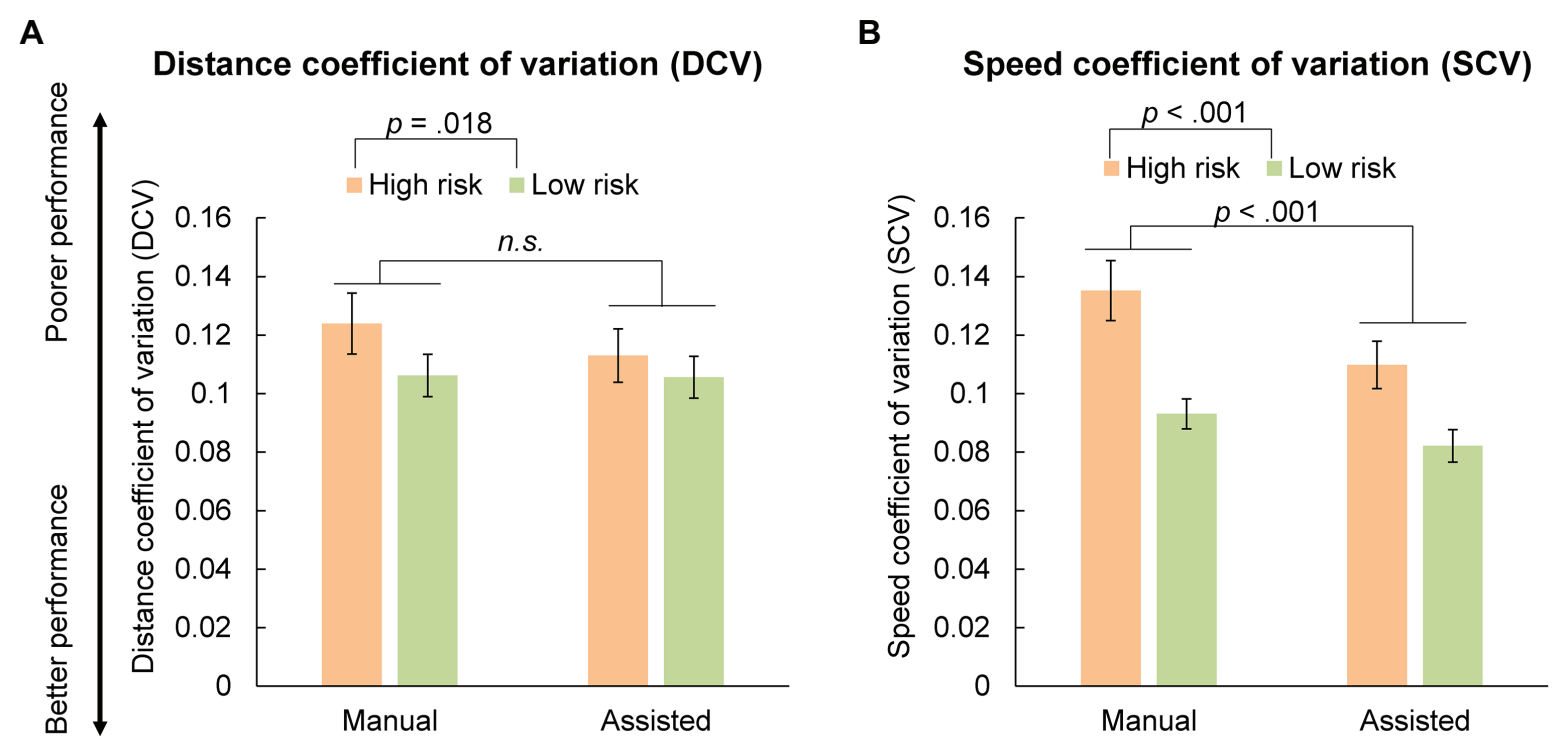
The average distance coefficient of variation (DCV; **A**) and the speed coefficient of variation (SCV; **B**). The error bars represent the SEs.

**Figure 4:**
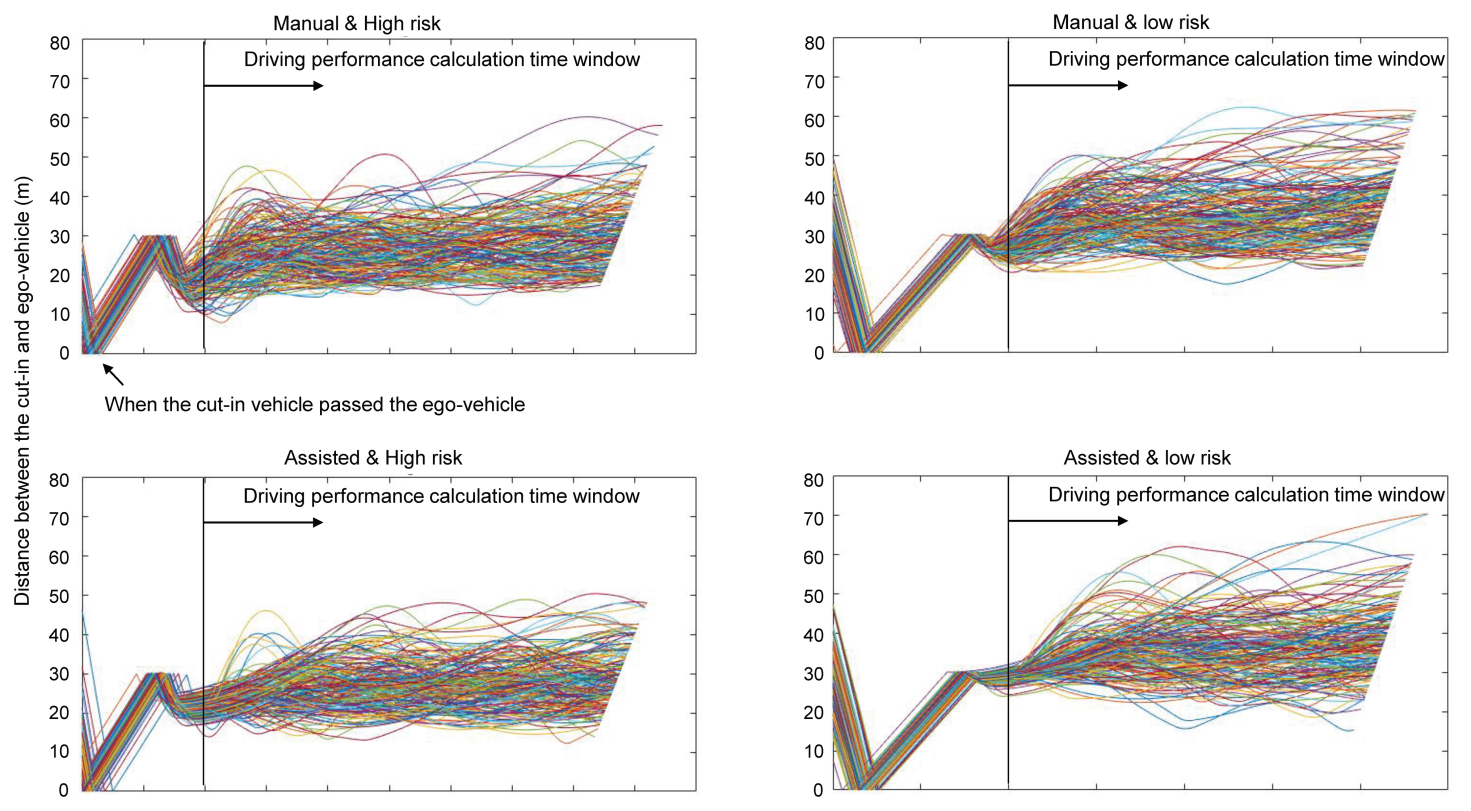
Plots of inter-vehicular distance in the calculation time window of each condition.

**Figure 5:**
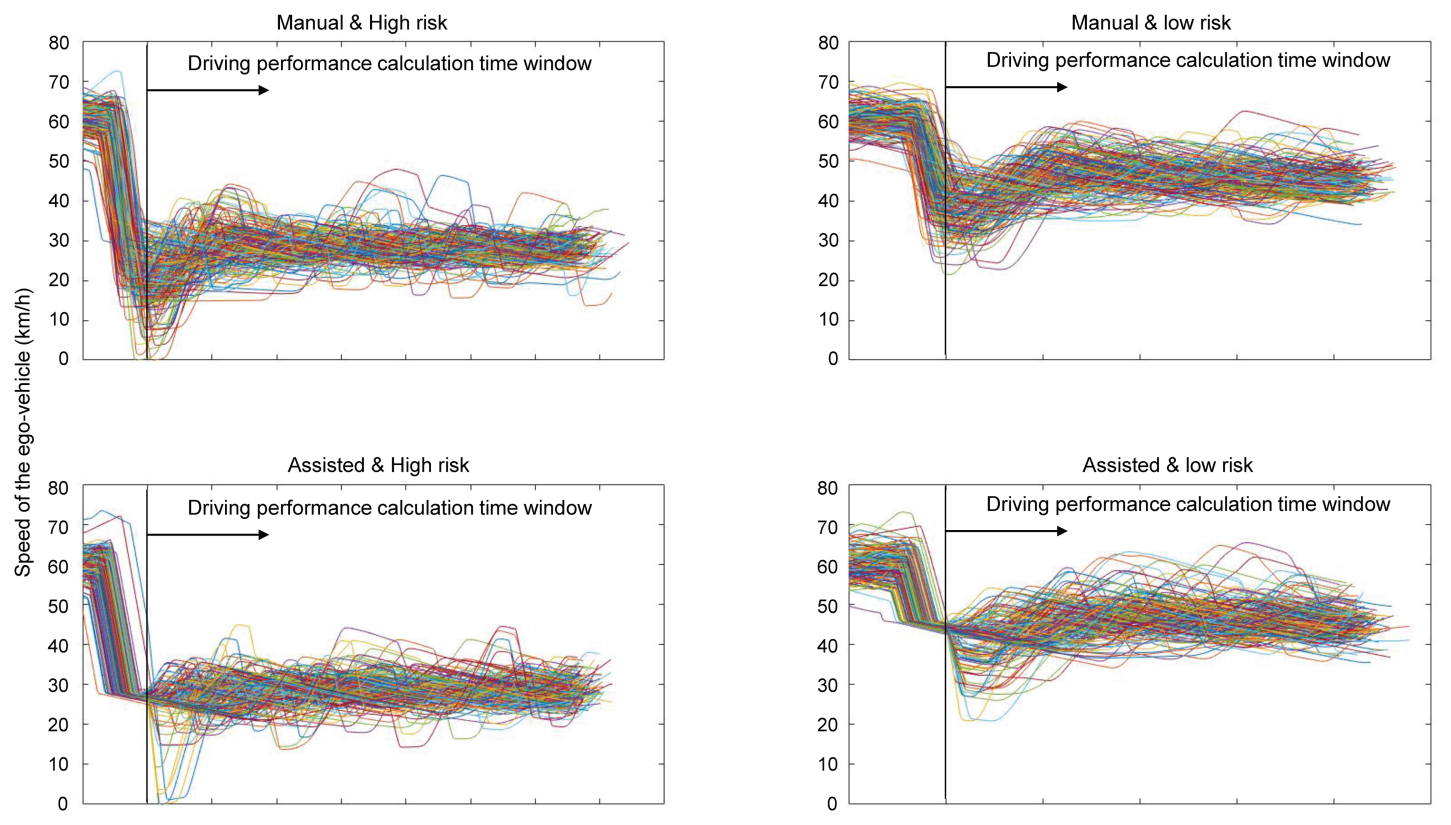
Plots of ego-vehicle speed in the calculation time window of each condition.

Figures 3A,B show the average value of DCV and SCV in each condition, indicating how well the inter-vehicle distance and speed were constantly maintained (a smaller value indicates better performance in both indices), respectively. Figure 4 plots the inter-vehicular distance and Figure 5 plots the speed of the ego-vehicle in each condition. Similar to the subjective rating, we used a 2 × 2 (driving mode: manual vs. assisted × risk: high vs. low) repeated measures ANOVA to examine the effect of the experimental conditions on each index of actual driving performance. Regarding the DCV (i.e., performance of inter-vehicle distance), the main effect of driving mode [*F*(1, 18) = 2.640, *p* = 0.122, *η*^2^_p_ = 0.128], and the interaction of driving mode and risk [*F*(1, 18) = 2.697, *p* = 0.118, *η*^2^_p_ = 0.130] were non-significant. However, the main effect of risk was significant [*F*(1, 18) = 6.731, *p* = 0.018, *η*^2^_p_ = 0.272]. The participants were better at maintaining a constant inter-vehicular distance in the low-risk condition than in the high-risk. This was probably because the ego-vehicle was closer to the cut-in vehicle during the emergency deceleration in the high-risk condition compared with the low-risk condition, resulting in greater difficulty in maintaining a constant inter-vehicular distance (Figure 4).

Regarding the SCV (i.e., performance of driving speed), the main effect of driving mode [*F*(1, 18) = 18.244, *p* < 0.001, *η*^2^_p_ = 0.503] and risk [*F*(1, 18) = 30.797, *p* < 0.001, *η*^2^_p_ = 0.631] were both significant. The interaction between driving mode and risk was non-significant [*F*(1, 18) = 2.649, *p* = 0.121, *η*^2^_p_ = 0.128]. The participants were better at maintaining a constant driving speed (following the cut-in vehicle) after receiving deceleration assistance and when the risk of collision was lower. Figure 5 showed that the assisted condition (i.e., lower panel) ensured smoother deceleration, consequently, resulting in smaller variation in speed after deceleration, compared to the manual condition (i.e., the top panel of Figure 5). In addition, no participants crashed into the cut-in vehicle during the experiment.

## Discussion

The present study examined the influence of an emergency automatic deceleration on sense of agency and driving performance. According to prior research, when driving assistance is activated, individuals usually feel a lower sense of control over their vehicle (Yun et al., 2018). The present study suggests that a driving assistance system that shares the same intention as the driver can eliminate the trade-off between sense of agency and driving performance. In the present study, participants decelerated their vehicles to avoid the risk of collision. The deceleration assistance ensured faster deceleration and, therefore, lowered their risks of accidents. The results showed that drivers maintained their sense of agency and improved their driving performance under deceleration assistance. Our findings provide useful insights into driving assistance design to address the trade-off between receiving driving assistance and maintaining driver engagement.

Previous empirical studies in cognitive psychology showed that sense of agency relies not only on the comparison between actions and sensory feedback (Carruthers, 2012; Synofzik et al., 2013; Wen et al., 2015a) but also greatly depends on task performance and goal achievement (Wen et al., 2015b; Inoue et al., 2017). However, when sensory feedback greatly deviates from one’s intentions or predictions, better task performance is seldom attributed to oneself, resulting in a reduced sense of agency. That is why driving assistance usually reduces sense of agency even though it ensures better driving performance (Yun et al., 2018). One important issue in the design of driving assistance is how to implement driving assistance without reducing sense of agency. Previous research provided a perspective on applying the theories and findings from cognitive science to the research on human-machine interfaces, highlighting the processing of shared intentions (Wen et al., 2019). The present study is the first to examine this research question in the context of driving: If the driving assistance technology does not conflict with a driver’s intentions, can it improve driving performance without reducing sense of agency? The present study showed that driving performance was indeed improved by deceleration assistance. It also indicated that subjective self-rating of driving performance also improved. Moreover, sense of agency remained at the same level between the manual and assisted driving conditions. In other words, the drivers accurately perceived that they performed better in the assisted driving condition, without realizing that the system actually took partial control over the vehicle.

It must be noted that the deceleration assistance used in the present study was very short (i.e., less than 4 s) and did not conflict with participants’ intentions. It is possible that the participants may have not noticed the driving assistance technology. However, the performance rating showed that participants indeed perceived the difference in the motion of the ego-vehicle between the manual and assisted conditions because they gave significantly higher ratings in the assisted condition. Furthermore, the deceleration assistance was only applied when the ego-vehicle was faster than the cut-in vehicle. That suggests that the assisted condition did not completely take over control of the vehicle, and participants’ level of control was still valid as long as there was no risk of collision. This probably also contributed to maintaining the sense of agency observed in the assisted condition.

The findings of the present study provide important insights for the future development of driving assistance functions and interfaces. Driving assistance technology that does not conflict with a driver’s intentions addresses the trade-off between sense of agency and driving performance. Human drivers feel that they are still in control of the vehicle and perform better with driving assistance. Therefore, it is critical to decode drivers’ intentions and employ assistance according to shared intentions (Kuge et al., 2000; Berndt and Dietmayer, 2009; Wang et al., 2018). In systems that need to propose driving strategies that conflict with driver intentions, it may be important to communicate the system’s intentions to drivers (Mulder et al., 2012). Some recent studies have shed light on the analysis of shared control conflicts and proposed conflict resolution frameworks to address this (Itoh et al., 2016; Flemisch et al., 2020). However, if the conflict resolution between a human and a system is not successful, the system should consider the decrease in sense of agency and propose a solution to recover the human’s sense of agency.

The present study emphasized the importance of shared intentions (i.e., absent of conflict) to address the trade-off between sense of agency and driving performance. A recent study suggested that both the initial holder of control (i.e., the agent currently with greater control of the vehicle) and intention consistency are important for the cooperative status between a human and a driving assistance system (Wada, 2019). These factors should all be considered in designing driving assistance. Furthermore, the active interventions of a driving assistance system can also result in skill improvements while reducing workload by providing guidance (Wada, 2019). In the present study, both self-rated driving performance and actual driving performance were improved by driving assistance. This indicates the possibility of skill development in driving using driving assistance without reducing sense of agency. Lastly, the improvements observed in driving performance in the assisted condition were not surprising and have been replicated in many studies on driving assistance (Young et al., 2011; Lyu et al., 2019; Saito et al., 2021). Importantly, our findings showed that well-tuned driving assistance is capable of making drivers attribute better driving performance to themselves.

There are some limitations to the present study. First, the deceleration assistance was specifically designed for the task to ensure no-conflict between the assists and drivers’ intentions. Therefore, our findings cannot generalize to all driving assistance types. Future research should be conducted to examine sense of agency and driving behaviors when a driving automation system proposes conflicting solutions to driver intentions. Second, the present study is still elementary on this topic. The essence of maintaining a sense of agency during driving assistance is the establishment of joint-control with a shared intention (Wen et al., 2019). The driving assistance provided in the present study was customized to cater to this demand. However, in reality, the system may not always be able to propose a solution that is congruent with drivers’ intentions due to safety reasons or human errors. Future research is needed to investigate and suggest more useful methods to establish joint-control based on shared intentions during driving assistance. In addition, the study was conducted in a driving simulator, which does not include motion information. The participants mainly relied on visual input and probably perceived less urgency in the near-crash condition. It is still unknown how driving assistance affects sense of agency and driving performance in a real driving environment. Lastly, all the participants in the present study were male, despite having no limit on participants’ gender during recruitment. This arguably limits the scope of our conclusions due to possible gender differences in sensitivity to external intervention, spatial cognition, sense of agency, and so on. Future studies with driving tasks need to balance participants’ genders to address this and reduce bias. To conclude, the present study highlighted an important direction in the development of driving assistance technologies, emphasizing the importance of considering drivers’ intentions and subjective feelings during driving assistance.

## Data Availability Statement

The raw data supporting the conclusions of this article has been deposited to Mendeley Data: http://dx.doi.org/10.17632/zvnybhpsry.1

## Ethics Statement

The studies involving human participants were reviewed and approved by the Ethics Committee of the School of Engineering at the University of Tokyo. The patients/participants provided their written informed consent to participate in this study.

## Author Contributions

WW and SY initiated and designed the research and analyzed the data. All authors reviewed the experimental design. SY performed the experiments. WW and BN wrote the manuscript. All authors contributed to the article and approved the submitted version.

### Conflict of Interest

BN was employed by the Toyota Research Institute.

The remaining authors declare that the research was conducted in the absence of any commercial or financial relationships that could be construed as a potential conflict of interest. The authors declare that this study received funding from Toyota Research Institute. The funder was not involved in the study design, collection, analysis, interpretation of data, the writing of this article, or the decision to submit for publication.
